# Effect of Osmolarity and Density of Colloid Formulations on the Outcome of SLC-Selection of Stallion Spermatozoa

**DOI:** 10.5402/2011/128984

**Published:** 2011-04-28

**Authors:** J. M. Morrell, A. Johannisson, H. Rodriguez-Martinez

**Affiliations:** Division of Reproduction, Department of Clinical Sciences, Swedish University of Agricultural Sciences (SLU), P.O. Box 7054, 75007 Uppsala, Sweden

## Abstract

The osmolarity and density of colloids used to prepare spermatozoa for assisted reproduction may affect sperm quality in the resultant preparation. In this study, two osmolarities of Androcoll-E for single-layer or density gradient centrifugation of stallion spermatozoa were compared: “normal” (320 mOsm) or “high” (345 mOsm). Mean yields for the two centrifugation techniques did not differ between treatments or osmolarities (single layer centrifugation: 30.19 ± 16.9 × 10^6^ and 25.8 ± 18.5 × 10^6^ spermatozoa; density gradient centrifugation: 31.84 ± 19.7 × 10^6^ and 26.46 ± 20.0 × 10^6^ spermatozoa respectively for the two osmolarities). However, use of the high osmolarity colloid for single layer centrifugation increased the proportion of morphologically normal spermatozoa (*P* < .05). Therefore, increasing the osmolarity of the colloid formulation may be beneficial for processing ejaculates containing a high proportion of abnormal spermatozoa by SLC. Reducing the density of the colloid used for the SLC substantially increased the yield of motile spermatozoa compared to the normal density colloid (mean ± SD: 72.6 ± 28.9 × 10^6^ versus 28.9 ± 24.7 × 10^6^), while also prolonging sperm survival by 24 hours compared to the uncentrifuged ejaculate. This increased yield may render Single Layer Centrifugation practical for use in the field.

## 1. Introduction

Over the past decade, the technique of centrifugation though a silane-coated silica colloid has been suggested as a means of preparing animal spermatozoa for artificial breeding [1, 2] reviewed by [3]. Density gradient centrifugation (DGC) has been used to prepare bovine spermatozoa for *in vitro* fertilization [4], to enhance stallion semen quality in preliminary studies [5], and, furthermore, both DGC and Single-Layer Centrifugation (SLC) have been employed to prepare stallion spermatozoa for diagnostics and for artificial insemination (AI) [6–8]. In the latter studies, the resulting sperm preparations showed an increase in normal morphology and chromatin integrity compared to the uncentrifuged sperm samples. However, despite the excellent quality of sperm preparations after colloid centrifugation, considerable numbers of spermatozoa are lost, a feature also seen with other sperm preparation methods, such as glass wool filtration, or swim-up (reviewed by [9]). This loss is a strong disincentive for the use of these preparation techniques in the artificial insemination (AI) industry. 

Earlier work on semen extenders indicated that sperm survival is benefited by using extenders with a higher osmolarity than animal semen [10]. In previous experiments with colloid centrifugation of boar spermatozoa at SLU, yields and quality were found to be improved by increasing the osmolarity of the colloid formulation from 305 mOsm to 330 mOsm (Morrell, unpublished data). Furthermore, preliminary studies with stallion spermatozoa showed that the quality of stallion sperm preparations could be improved by increasing the osmolarity of the colloid from 305 to 320 mOsm. Reducing the density of the colloid should increase the numbers of spermatozoa passing through during centrifugation, although the selection process might be impeded or diminished, resulting in poorer quality sperm preparations. However, it might be possible to strike a balance between sperm numbers and quality, to obtain sufficient spermatozoa after SLC for conventional AI. 

The objective of the present study was to determine the effects of osmolarity and density of colloid formulations on the quality of sperm selection by centrifuging stallion spermatozoa through colloid formulations with osmolarities of 320 mOsm (“normal”) and 345 mOsm (“high”), and by using lower-density formulations.

## 2. Material and Methods

### 2.1. Animals and Husbandry

- A warmblood stallion of breeding age but unknown fertility was housed under standard husbandry conditions at the Division of Reproduction, SLU, Uppsala. Semen was collected up to 1-2 times a week over a 6-week period during the normal breeding season (April to August in Sweden) using an artificial vagina *as praxis*. Material not required for teaching purposes was made available for the experiments.
- Warmblood stallions of breeding age (7–23 years) were housed under standard husbandry conditions at Flyinge AB, Flyinge, Sweden. Semen was collected up to three times a week during the normal breeding season as part of a commercial enterprise. The ejaculates were extended 1 : 1 with either Kenney's extender (1975) or Nørlunds media (see below) at 37°C. Aliquots (6 mL) of the extended ejaculates were made available for a comparison of the two methods of colloid centrifugation.

### 2.2. Media

Kenney's extender [11]: to one deciliter of water were added glucose (4.9 g), skimmed milk powder (2.4 g), dihydrostreptomycin (0.15 g), and penicillin (0.15 g).

Nørlunds extender: this commercially available medium was purchased from Nørlunds Equine Hospital, Rue de Lund, 8653 Them, Denmark. 

Colloid: silane-coated silica in a buffered salt solution (Androcoll-E; patent applied for) was used for the density gradient (DGC) and single-layer centrifugation (SLC). Two densities were used for the upper and lower layers of the DGC, and various densities were used for the SLC (see below for details).

### 2.3. Sperm Concentration

The concentration of spermatozoa in the original ejaculate was measured using a Spermacue photometer. The sperm concentration of the extended semen was counted using a Bürker counting chamber and adjusted to approximately 100 × 10^6^/mL where necessary.

### 2.4. Density Gradient Centrifugation

A density gradient was prepared by pipetting 2 mL colloid of density 1.104 g/mL into a centrifuge tube and carefully layering 2 mL colloid of density 1.052 g/mL on top; an aliquot (1.5 mL) of extended semen containing approximately 100 × 10^6^ spermatozoa/mL was pipetted on top of the upper layer. The gradient was centrifuged at 300 × g for 20 minutes, after which the diluted seminal plasma and most of the gradient material containing spermatozoa that had not passed all the way through the colloid were discarded. The sperm pellet was transferred to a clean centrifuge tube containing 5 mL Kenney's extender and was washed by centrifuging for 10 minutes at 500 × g. Following washing, the sperm pellet was resuspended in fresh Kenney's extender (1 mL).

### 2.5. Single-Layer Centrifugation

The method was similar to that for DGC with the exception that 4 mL of the higher density material (= normal density, 1.104 g/mL) was pipetted into the centrifuge tube instead of two layers of different densities (2 mL of each density). For the experiment with different colloid densities, a single layer of colloid of density 1.052 g/mL or 1.078 g/mL was used.

### 2.6. Subjective Estimation of Motility

Aliquots (0.5 *μ*L) of the extended ejaculate and sperm preparations were examined by phase contrast light microscopy (×200), on a heated microscope stage (38°C) immediately after preparation and once daily until the motility had dropped to approximately 20%.

### 2.7. Sperm Morphology

Smears of extended ejaculates and the sperm preparations were made on clean glass slides. In addition, a few drops of each sperm suspension were added to buffered formaldehyde [12]. The staining and evaluation have been described in detail [7]. The mean proportion of morphologically normal spermatozoa was estimated as the remaining proportion left from total abnormal spermatozoa counted both in wet smears and Williams stained slides (100-total abnormalities). Note “normal morphology” was taken to be the percentage of normal spermatozoa from the formol saline count plus the percentage of spermatozoa with distal cytoplasmic droplets.

### 2.8. Sperm Chromatin Structure Assay

Aliquots (0.5 mL) of the extended semen and sperm preparations were mixed with an equal volume of Tris-NaCl-Ethylenediaminetetra-acetic acid (TNE) buffer and frozen immediately in liquid nitrogen. The samples were stored in liquid nitrogen vapour for up to three weeks before being transferred to a freezer at −80°C until required for analysis by flow cytometry. 

Abnormal chromatin structure was defined as the susceptibility of sperm DNA to undergo acid-induced denaturation *in situ*. Following exposure of the prepared DNA to acridine orange (AO), the degree of chromatin integrity was analysed by flow cytometric measurement of the metachromatic shift from green (stable, double-stranded DNA) to red (denatured, single-stranded DNA) AO fluorescence [13]. This shift was expressed as %DFI (DNA fragmentation index) which shows the ratio of red to total (i.e., red and green) fluorescence intensity. In the SCSA, DFI was calculated for each spermatozoon within a sample, and the results were expressed as the mean (X_DFI), the standard deviation of the distribution (SD_DFI) [14]. 

In the present study, the procedure originally developed by Evenson et al. [13] and later described in detail by [15] was used.

### 2.9. Experimental Design

#### 2.9.1. Experiment 1: Effect of Different Osmolarities of Colloid

Density gradients and single layers were prepared using colloid preparations of different osmolarities. Ejaculates from one stallion (*n* = 5) were used to compare two osmolarities (300–310 and 330 mOsm), whereas 12 ejaculates from 10 stallions were used to compare two osmolarities (320 and 345 mOsm, termed “normal” and “high,” resp.). The resulting sperm preparations were assessed for sperm concentration and sperm motility, as described previously while, in addition, samples in the larger trial were collected for morphological and chromatin analysis.

#### 2.9.2. Experiment 2: Comparison of Different Densities for SLC

Spermatozoa were prepared by SLC using colloid of density 1.104 g/mL, corresponding to the density used for the bottom layer in a DGC, and another, lower, density of 1.052 g/mL (corresponding to the upper layer used in the DGC) (*n* = 3 ejaculates). This experiment was repeated using a single layer of an intermediate density, 1.078 g/mL (*n* = 8). Sperm concentration and motility were assessed on a daily basis until motility dropped to 20% as previously described.

#### 2.9.3. Statistics

The mean values of yield, proportion of morphologically normal spermatozoa and %DFI in the uncentrifuged and SLC sperm samples were analysed by Analysis of Variance, (PROC MIXED) using the Statistical Analysis Software (SAS version 9; SAS Institute Inc, Cary, NC, USA). In all cases, *P* < .05 was used to denote a significant difference.

## 3. Results

### 3.1. Experiment 1

**Figure 1:**
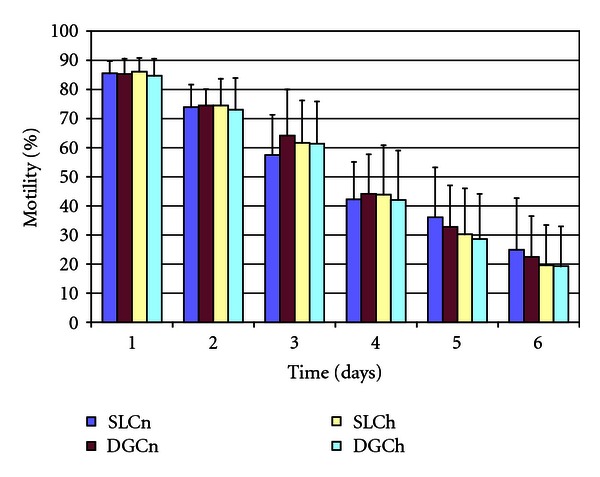
Effect of osmolarity of colloid formulation on sperm motility (mean ± SD) with time after colloid centrifugation (*n* = 15). SLC: single-layer centrifugation, DGC: gradient density centrifugation, n: normal osmolarity colloid (320 mOsm), h: high osmolarity colloid (345 mOsm), and Day 1: day of semen collection.

The effect of osmolarity of the colloid on the yield of motile spermatozoa obtained after centrifugation on a colloid is shown in Table 1. The mean numbers of spermatozoa harvested from the colloids with different osmolarities were not statistically different, although there were considerable differences between individuals (*P* < .001). Sperm survival did not differ between the colloid treatments (Figure 1). 

**Figure 2:**
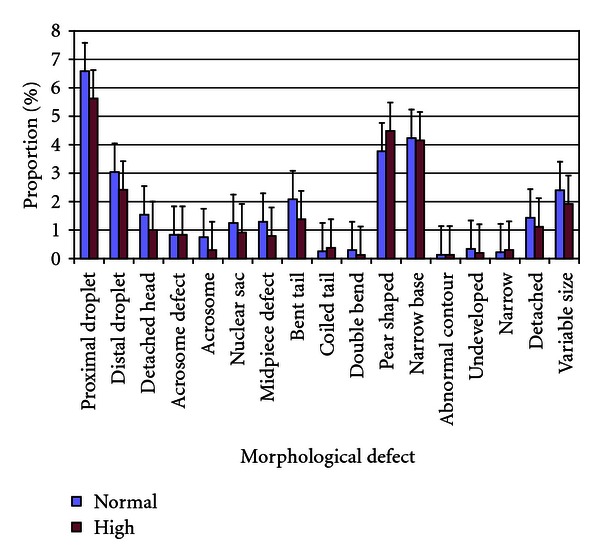
Effect of colloid osmolarity (320 mOsm (normal) and 345 mOsm (high)) on the proportion of morphological abnormalities observed among stallion spermatozoa (*n* = 12).

There were significant differences between the controls (uncentrifuged) and the treatment groups for normal morphology (*P* < .001) and %DFI (*P* < .001) (Table 2). Use of a high osmolarity colloid for the SLC resulted in a significant increase in the number of morphologically normal spermatozoa compared with SLC normal (*P* < .05) and DGC normal (*P* < .05), and also a numerical increase over DGC high, with a trend towards significance (*P* < .09). For individual morphological abnormalities, differences in the ability of the normal and high osmolarity colloid formulations to remove abnormal spermatozoa were not significant (Figure 2).

### 3.2. Experiment 2

**Figure 3:**
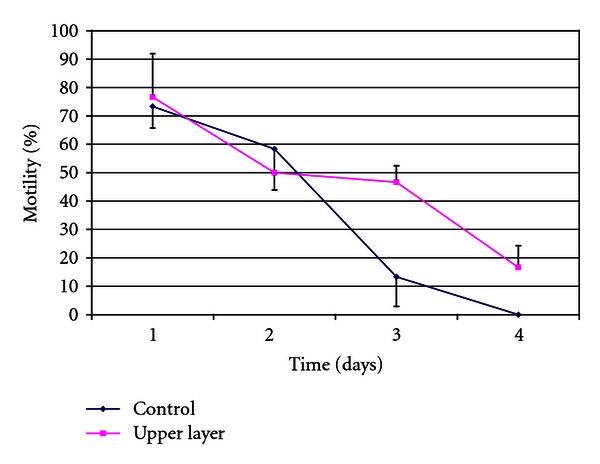
Effect on sperm survival of separation of spermatozoa from seminal plasma by a colloid, without any selection for good quality spermatozoa (*n* = 3). Control: uncentrifuged spermatozoa, upper layer: single-layer centrifugation through Androcoll-E of density 1.052 g/mL, equivalent to the upper layer of a density gradient.

**Figure 4:**
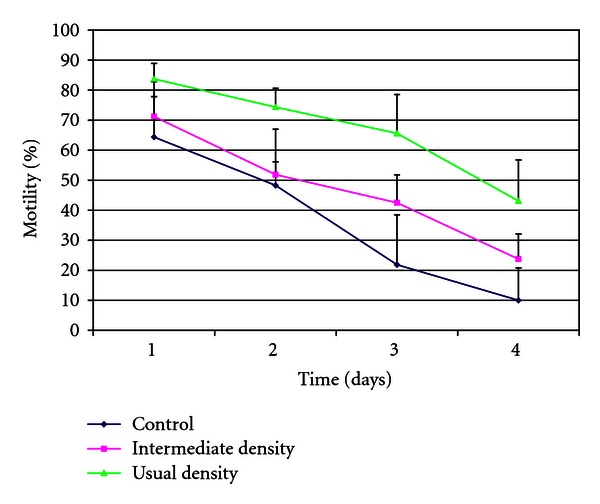
Comparison of different densities of colloid on stallion sperm motility after single-layer centrifugation (*n* = 8). Control: uncentrifuged spermatozoa, usual density: single-layer centrifugation through Androcoll-E of density 1.104 g/mL, intermediate density: single-layer centrifugation through Androcoll-E of density 1.078 g/mL.

After centrifugation through an SLC of low density, the sperm preparations were very similar to the control (uncentrifuged semen) in terms of numbers of motile spermatozoa, presence of cellular debris, and so forth. However, sperm survival was extended by approximately 24 hours in the centrifuged preparations (Figure 3). When the spermatozoa were prepared on an SLC of an intermediate density, the preparations contained a higher proportion of motile spermatozoa, and these spermatozoa survived longer than controls (uncentrifuged aliquot), although neither parameter was as good as in the samples prepared on the normal single layer (Figure 4). Sperm numbers were significantly higher (*P* < .01) for the samples prepared on the intermediate density colloid compared to the usual single layer (mean ± SD: 72.6 ± 28.9 million compared to 28.9 ± 24.7 million).

## 4. Discussion

In the present study, where stallion spermatozoa were prepared on colloid formulations with different osmolarities, there was a trend for the yield of spermatozoa recovered to decrease as osmolarity increased, although these differences were not significant. There were also differences between stallions and between ejaculates from individual stallions, confirming previous reports of large variations in quality between stallions and between ejaculates. Therefore, inter-ejaculate and inter-individual differences may have masked an effect due to the osmolarity of the colloid. 

The observation that removal of morphologically abnormal spermatozoa could be enhanced by increasing the osmolarity of the colloid is interesting and warrants further investigation, particularly for stallions with a specific morphological abnormality. The removal of spermatozoa with most types of morphological defects was enhanced by increasing the osmolarity of the colloid, with the exception of pear-shaped heads. Abnormal head shapes could be expected to alter sperm density, for example, by increasing the sperm mass while maintaining volume, leading to an increase in density [7]. Such spermatozoa would pellet ahead of their normal counterparts. 

Our results also show that it is possible to reduce sperm losses during colloid centrifugation by altering the density of the colloid used. In the experiment reported here, simply reducing the density of the SLC resulted in a significantly larger yield of motile spermatozoa in the preparation, with longer sperm survival than in the uncentrifuged ejaculate, although sperm motility was not retained for as long as when a colloid of normal density was used for the centrifugation. This result is interesting and warrants further investigation. It is possible that the quality of the sperm preparations from the reduced density SLC would be of sufficient quality for their immediate use in insemination or cryopreservation. 

If these preliminary results are confirmed, this method could suggest an alternative preparation technique whereby sperm quality could be enhanced and sperm survival increased by 24 hours compared to uncentrifuged ejaculates, without the large loss of spermatozoa seen with SLC using the normal (higher) density colloid. Furthermore, SLC could facilitate the preparation of spermatozoa for freezing, surpassing the use of simple centrifugation of extended semen to remove the bulk of the seminal plasma and the extender. Note that the choice of preparation method for cryopreserving stallion spermatozoa is somewhat surprising, since studies with human spermatozoa have shown that centrifuging spermatozoa in seminal plasma without the presence of a colloid results in generation of reactive oxygen species (ROS) which are detrimental to sperm survival. If the same phenomenon is observed for stallion spermatozoa during centrifugation without a colloid, the generation of ROS during preparation for freezing could help to explain the variable survival currently obtained when attempting cryopreservation in this species. 

In conclusion, increasing the osmolarity of the colloid formulation up to 345 mOsm improves the quality of the sperm preparation, but does not affect sperm chromatin integrity. However, increasing the osmolarity above 320 mOsm may decrease sperm yield. Therefore, a possible improvement in sperm quality must be balanced against a tendency for decreased yield. The yield of stallion spermatozoa can be increased by lowering the density of the colloid used for SLC.

## Figures and Tables

**Table 1:** Effect of osmolarity of colloid on mean (±SD) number of motile spermatozoa in the pellet after single-layer centrifugation (*n* = 20).

Normal osmolarity (305–320 mOsm)		High osmolarity (330–345 mOsm)	
SLC	DGC	SLC	DGC
30.19 × 10^6^	31.84 × 10^6^	25.8 × 10^6^	26.46 × 10^6^
±16.9	±19.7	±18.5	±20.0

SLC: single-layer centrifugation, DGC: gradient density centrifugation.

**Table 2:** Effect of osmolarity of colloid on normal sperm morphology and %DNA fragmentation index (mean ± SD) (*n* = 12).

Parameter	Control	SLC normal	SLC high	DGC normal	DGC high
Normal morphology (%)	68.2 ± 14.8^a^	78 ± 7.9^ab^	82.8 ± 9.0^ab^	78 ± 8.2^ab^	79.4 ± 8.1^a^
%DFI	12.4 ± 4.4^a^	6.4 ± 5.4^a^	5.9 ± 4.2^a^	5.3 ± 3.0^a^	5.0 ± 4.3^a^

^a^Control significantly different to other treatments *P* < .001; ^b^SLC high > SLC normal and SLC high > DGC normal (*P* < .05 for both). SLC: single layer centrifugation, DGC: gradient density centrifugation.

## References

[1] Rodriguez-Martinez H, Larsson B, Pertoft H (1997). Evaluation of sperm damage and techniques for sperm clean-up. *Reproduction, Fertility and Development*.

[2] Hallap T, Håård M, Jaakma Ü, Larsson B, Rodriguez-Martinez H (2004). Does cleansing of frozen-thawed bull semen before assessment provide samples that relate better to potential fertility?. *Theriogenology*.

[3] Morrell JM (2006). Update on semen technologies for animal breeding. *Reproduction in Domestic Animals*.

[4] Samardzija M, Karadjole M, Matkovic M (2006). A comparison of BoviPure® and Percoll® on bull sperm separation protocols for IVF. *Animal Reproduction Science*.

[5] Morrell JM, Dalin A-M, Rodriguez-Martinez H (2008). Prolongation of stallion sperm survival by centrifugation through coated silica colloids: a preliminary study. *Animal Reproduction*.

[6] Macpherson M, Blanchard TL, Love CC, Brinsko SP, Thompson JA, Varner DD (2003). *Proceedings of the 49th Annual Convention of the American Association of Equine Practitioners*.

[7] Morrell J, Johannisson A, Dalin A-M, Rodriguez-Martinez H (2009). Morphology and chromatin integrity of stallion spermatozoa prepared by density gradient and single layer centrifugation through silica colloids. *Reproduction in Domestic Animals*.

[8] Morrell JM, Dalin AM, Rodriguez-Martinez H (2009). Comparison of density gradient and single layer centrifugation of stallion spermatozoa: yield, motility and survival. *Equine Veterinary Journal*.

[9] Sieme H, Martinsson G, Rauterberg H (2003). Application of techniques for sperm selection in fresh and frozen-thawed stallion semen. *Reproduction in Domestic Animals*.

[10] Watson PF, Lamming GE (1990). Artificial insemination and the preservation of semen. *Marshall’s Physiology of Reproduction*.

[11] Kenney RM, Bergman RV, Cooper WL, Morse GW (1975). Minimal contamination techniques for breeding mares: techniques and preliminary findings. *Proceedings of American Association of Equine Practitioners*.

[12] Hancock L (1952). The morphology of bull Spermatozoa. *British Journal of Experimental Biology*.

[13] Evenson DP, Darzynkiewicz Z, Melamed MR (1980). Relation of mammalian sperm chromatin heterogeneity to fertility. *Science*.

[14] Evenson DP, Larson K, Jost LK (2002). The sperm chromatin structure assay (SCSATM): clinical use for detecting sperm DNA
fragmentation related to male infertility and comparisons with other techniques. *Journal of Andrology*.

[15] Johannisson A, Morrell JM, Thorén J, Jönsson M, Dalin AM, Rodriguez-Martinez H (2009). Colloidal centrifugation with Androcoll-E*™* prolongs stallion sperm motility, viability and chromatin integrity. *Animal Reproduction Science*.

